# Component Processes of Decision Making in a Community Sample of Precariously Housed Persons: Associations With Learning and Memory, and Health-Risk Behaviors

**DOI:** 10.3389/fpsyg.2021.571423

**Published:** 2021-07-02

**Authors:** Heather A. Baitz, Paul W. Jones, David A. Campbell, Andrea A. Jones, Kristina M. Gicas, Chantelle J. Giesbrecht, Wendy Loken Thornton, Carmelina C. Barone, Nena Y. Wang, William J. Panenka, Donna J. Lang, Fidel Vila-Rodriguez, Olga Leonova, Alasdair M. Barr, Ric M. Procyshyn, Tari Buchanan, Alexander Rauscher, G. William MacEwan, William G. Honer, Allen E. Thornton

**Affiliations:** ^1^Department of Psychology, Simon Fraser University, Burnaby, BC, Canada; ^2^Department of Psychiatry, University of British Columbia, Vancouver, BC, Canada; ^3^British Columbia Mental Health and Substance Use Services, Research Institute, Vancouver, BC, Canada; ^4^Department of Statistics and Actuarial Science, Simon Fraser University, Burnaby, BC, Canada; ^5^School of Mathematics and Statistics, Carleton University, Ottawa, ON, Canada; ^6^Department of Psychology, York University, Toronto, ON, Canada; ^7^Department of Radiology, University of British Columbia, Vancouver, BC, Canada; ^8^Department of Anesthesiology, Pharmacology, and Therapeutics, University of British Columbia, Vancouver, BC, Canada; ^9^Department of Pediatrics, University of British Columbia, Vancouver, BC, Canada

**Keywords:** decision making, Iowa Gambling Task, substance use, marginalization, precariously housed, health risk, prospect valence learning model, homelessness and housing

## Abstract

The Iowa Gambling Task (IGT) is a widely used measure of decision making, but its value in signifying behaviors associated with adverse, “real-world” consequences has not been consistently demonstrated in persons who are precariously housed or homeless. Studies evaluating the ecological validity of the IGT have primarily relied on traditional IGT scores. However, computational modeling derives underlying component processes of the IGT, which capture specific facets of decision making that may be more closely related to engagement in behaviors associated with negative consequences. This study employed the Prospect Valence Learning (PVL) model to decompose IGT performance into component processes in 294 precariously housed community residents with substance use disorders. Results revealed a predominant focus on gains and a lack of sensitivity to losses in these vulnerable community residents. Hypothesized associations were not detected between component processes and self-reported health-risk behaviors. These findings provide insight into the processes underlying decision making in a vulnerable substance-using population and highlight the challenge of linking specific decision making processes to “real-world” behaviors.

## Introduction

Persons trapped in unstable and impoverished housing endure remarkable burdens including health and substance use challenges, compromised safety within the context of victimization, social isolation, stigma, and barriers that hinder access to essential services (Kushel et al., 2002, 2003; Shannon et al., 2006). Indeed, precariously housed “hotel” residents living in single-room occupancy (SRO) accommodations in Vancouver, British Columbia suffer from high rates of substance dependence and a plethora of other physical and mental illnesses (Vila-Rodriguez et al., 2013). Furthermore, these persons have a standardized mortality ratio eight times greater than expected based on age- and sex-matched Canadian population data (Jones et al., 2015).

Substance use may occur or increase following deterioration of health, financial burden, loss of employment, and estrangement from family and friends. Increasing use often exacerbates these problems (Sinha, 2008). Chronic use is associated with a multitude of serious personal and societal consequences. Continued substance use despite significant distress and life problems is a core feature of substance use disorders (American Psychiatric Association, 2013). Dependence entails a neurobiologically mediated diminished ability to inhibit a behavior despite serious adverse consequences (Volkow et al., 2014). This manifestation can be conceptualized as impairment in decision making, defined as continuing to choose options that maximize short-term benefits but lead to long-term negative outcomes (Bechara, 2003). Conversely, along with adequate resources and treatments, decisions that prioritize long-term benefits are necessary for lifestyle changes and treatment success (Stevens et al., 2014).

Daily behaviors carrying risk of harm are ubiquitous in humans (Frankenhuis and Del Giudice, 2012). For example, millions of people drive motor vehicles despite fatal accidents. The evolutionary perspective suggests that higher risk levels are accepted when stakes are higher (e.g., animals close to starvation should forage even when the risk of being caught by a predator is high; Frankenhuis and Del Giudice, 2012). It may be that decisions taken by impoverished persons with ever-present security concerns are substantially compromised by neurobehavioral dysfunction arising in the interplay between daily substance use, evolving substance use disorders, and underlying brain pathologies (e.g., traumatic and cerebrovascular; Schmitt et al., 2017; Zhou et al., 2019, 2020; Stubbs et al., 2020). Precariously housed persons may experience particularly negative consequences for decisions arising in the context of structural vulnerabilities when combined with loss of agency that limits options to those that may entail higher risks (McNeil et al., 2015; e.g., mandated changes in methadone treatment regimens may produce access barriers that are mitigated by accepting choices involving higher personal risks). To reduce barriers within a harm-reduction framework, public health research is increasingly focused on social and structural vulnerabilities (e.g., environment’s impact on individual, community, and policy interventions: Rhodes, 2002; McNeil et al., 2015).

Nonetheless, individual factors impacting decision making are essential to understand. Prior work reveals that the trait of sensation seeking, which involves a willingness to take risk to manipulate arousal and elicit sensations (Zuckerman, 1994), is associated greater substance use (e.g., Ripa et al., 2001; Hittner and Swickert, 2006) and more hazardous substance use practices. Indeed, greater sensation seeking tendencies have been associated with increased needle injection, methamphetamine use, binging, and polysubstance use in street-involved youth (Werb et al., 2015).

Laboratory-based decision making tasks have also been developed for the very purpose of assessing decision making when outcome probabilities for risks are complex and uncertain, as they often are in life. The Iowa Gambling Task (IGT; Bechara et al., 1994) closely maps behaviors that are reliably rewarding but may lead to occasional negative consequences (Mukherjee and Kable, 2014). Participants choose cards from four decks. To succeed, draws must be made more often from the low- versus high-risk decks, the latter of which have higher payoffs but ultimately even higher losses. Perhaps because of its verisimilitude appeal and apparent sensitivity, the IGT has been widely adopted by researchers interested in risk behaviors in the real world.

Indeed, the IGT detects impairments of similar magnitude across various mental illnesses, with moderate impairment observed in samples of persons suffering from substance dependence (Hedges’ *g* = 0.63; Mukherjee and Kable, 2014; also see Kovács et al., 2017). Investigators have also examined the conditions and extent to which “risky” IGT card selections signify a propensity to make potentially hazardous decisions in daily life. “Poorer” performance on the task predicts initiating ecstasy use in women but not men (Schilt et al., 2009), high levels of binge alcohol use (Goudriaan et al., 2007) and abstinence (Stevens et al., 2014, 2015). Nonetheless, larger studies report null main effect associations between IGT and high-risk sexual encounters in HIV seropositive men (Gonzalez et al., 2005; Wardle et al., 2010) and/or conditionalized effects with interactions emerging in accordance with quantity and nature of emotional distress (e.g., absent, anxiety, and depression) or the risk behaviors examined (Wardle et al., 2010; Golub et al., 2016). Indeed, better IGT performance has occasionally been selectively associated with more engagement in hazardous behaviors. Notably, there is a relative absence of decision making investigations of actively using populations who encounter health hazards based upon the substances used, quantities, administration routes, and associated risk behaviors (needle or pipe sharing). Interestingly, in a study conducted in a university setting, no overall group differences in IGT decision making were apparent between students who use primarily cannabis and alcohol compared to their controls. Surprisingly, gender moderated the effect. Only substance using males exhibited IGT impairments in a pattern indicating that these men attended to card wins over losses (Stout et al., 2005).

Some potential features of past work contributing to mixed findings include (a) reduced IGT score variance arising from stricter exclusion criteria^1^, (b) frequent use of abstinent samples facing no ongoing substance acquisition or use hazards (e.g., opioid use) thereby narrowing sampling of potentially hazardous behavior, and (c) reliance on traditional IGT Net scores that do not capture the specific cognitive, motivational, and response processes underlying decision making. In an actively using community sample, we report on links between decision making processes derived from the IGT and health-risk behaviors that have potential for long-term adverse consequences. Our aim is to evaluate the utility of the IGT in signifying behaviors that entail health risks within an ecologically valid context. We target substance use and associated health behaviors as they naturalistically occur in the community. To capture specific cognitive, motivational, and response processes, we used computational modeling, which is an increasingly popular tool for decomposing IGT processes (Busemeyer and Stout, 2002; Bull et al., 2015; Kildahl et al., 2020).

To capture IGT-delineated decision making we utilize the Prospect Valence Learning (PVL) model, an accurate, generalizable model that includes four parameters representing distinct processes (Ahn et al., 2008; Fridberg et al., 2010). The utility function in the PVL model, derived from prospect theory (Tversky and Kahneman, 1992), accounts for the commonly observed preference for decks with less frequent losses. The *attention to losses parameter* reflects the relative weights given to amounts lost versus amounts gained. The *attention to magnitude parameter* reflects the extent to which the subjective value of an outcome is proportional to its magnitude. When there is no attention to magnitude, all losses are treated as equally bad and all gains are treated as equally good. Participants’ expectations about the outcomes they are likely to obtain (“expected values”) are calculated based on a weighted combination of the most recent outcome and more distant previous outcomes. The weight given to the most recent outcome versus previous outcomes is determined by the *retention parameter*. Shorter retention indicates more consideration of the most recent outcome and less consideration of more distant outcomes, i.e., faster updating. The *consistency parameter* represents how closely choices align with the highest expected deck value.

The attention to losses parameter captures the most promising component process in signifying health-risk behavior, given the parallel weighing of rewards and consequences involved in decision making outside the laboratory. Our primary hypothesis was that within an actively substance using, precariously housed population, those persons exhibiting lower attention to losses would engage in more frequent behaviors that are hazardous to health (Hypothesis 1). Persons who pay relatively more attention to gains and less attention to losses in the laboratory may experience high sensitivity to reward or low sensitivity to negative consequences in their daily lives. Indeed, by diagnoses, persons with substance use disorders persist in use despite negative consequences. These individuals generally demonstrate low attention to losses on the IGT (Stout et al., 2004, 2005; Yechiam et al., 2005; Fridberg et al., 2010; Vassileva et al., 2013; Ahn et al., 2014; Krmpotich et al., 2015; but also see Sevy et al., 2007; Bishara et al., 2009; Lane et al., 2010 for null findings, yet in the expected direction).

We further hypothesized that shorter retention (i.e., faster updating) would be associated with poorer learning and memory in a cognitively impaired community sample (see Gicas et al., 2014; Hypothesis 2). This prediction is consistent with the observation that acute administration of benzodiazepines, which are memory antagonists (Ghoneim and Mewaldt, 1990; Lundqvist, 2005), cause shorter retention (Lane et al., 2006, 2008). Shorter retention involves more consideration of the most recent and less consideration of distant outcomes, which might occur if a decision maker (a) is unable to learn and remember distant outcomes and/or (b) responds impulsively, i.e., without consideration of distant outcomes.

The associations between the IGT parameters and frequency of use of specific substances were explored to illuminate any selective associations between decision making processes and substance use frequency. In addition, we explored whether the IGT parameters were associated with sensation seeking as well as the extent to which sensation seeking was associated with health-risk behaviors. For completeness, we also evaluated the associations of conventional IGT Net scores with health-risk behavior, learning and memory ability, and sensation seeking.

## Methods

### Participants

The overall, comprehensive study of health and comorbidity in people living in precarious housing was approved by the Clinical Research Ethics Board (REB) of the University of British Columbia and the REB at Simon Fraser University; the latter also approved this specific project. Consenting participants, able to communicate in English, were recruited between November 2008 and August 2012 from SRO hotels and the community court in a low-income neighborhood as part of a longitudinal study (“Hotel Study;” see Vila-Rodriguez et al., 2013). Participants subsequently attend monthly follow-up sessions for up to ten years and receive modest cash honorariums. IGT data was collected at the baseline time point for 327 participants in the longitudinal study (275 recruited from SRO hotels and 52 recruited from community court). For the present analyses, we included only participants with valid IGT data (see below) and well-documented lifetime and current histories of substance use and associated health-risk behaviors.

Under the approval of the REB at Simon Fraser University, an additional sample of 138 volunteers was recruited (122 undergraduates and 16 participants responding to our advertisement) for the primary purpose of ensuring modeling robustness. Hereafter, these participants are referred to as the “calibration” sample. This sample differs from the community sample in several important ways, including the presumed absence of serious substance use disorder and their lack of exposure to precarious housing, as well as in education, age, gender, physical and mental health, and family background. Calibration participants attended one session and received course credit or a modest cash honorarium.

Characteristics of the final community sample (*n* = 294; 78.9% male) and the final calibration sample (*n* = 136; 30.1% male) with valid data^2^ are presented in Table 1. We did not attempt to match these groups in our research design, or to statistically control for group differences, given that our primary interest was in understanding the utility of the IGT in identifying individual factors associated with health-related risk-taking behaviors. Further, a true control group entails challenges given the population’s developmental uniqueness and a limited capacity to control for numerous variables potentially impacting IGT performance (e.g., brain injury, chronic viral infections, severe mental illnesses, developmental disorders, and their interactions) in a group comparison design.

**TABLE 1:** Characteristics of the community and calibration samples.

Characteristic	Community sample				Calibration sample		
	%	Mean (SD)	Median	Range	Mean (SD)	Median	Range
Age (years)		43.1 (9.5)	44.0	23-68	21.8 (6.5)	20.0	17-52
Education (years)^a^		10.2 (2.3)	10.0	2-16	13.0 (1.3)	13.0	10-18
Premorbid IQ (WTAR estimate)^b^		96.4 (9.6)	97.0	70-122	103.6 (8.3)	105.0	77-121
Role functioning (RFS)^c^		11.9 (3.3)	12.0	5-24			
Global functioning (GAF)^d^		38.2 (10.4)	37.0	15-70			
**Ethnic origin**							
European	59.6						
Indigenous	27.7						
Other	12.7						
**Psychiatric diagnosis**							
Schizophrenia spectrum	12.9						
PNOS	12.6						
Major depression	14.7						
Bipolar disorder I or NOS	5.4						
Bipolar disorder II	2.4						
Substance induced disorders	29.6						
**Substance dependence (baseline)**							
Alcohol	18.0						
Cannabis	32.7						
Cocaine	69.0						
Methamphetamine	25.5						
Heroin	36.9						
HIV seropositivity	15.2						
HCV antibody reactivity	62.2						

*Higher GAF and RFS scores indicate better functioning. WTAR = Weschler Test of Adult Reading; RFS = Role Functioning Scale; GAF = Global Assessment of Functioning; PNOS = Psychosis Not Otherwise Specified; NOS = Not Otherwise Specified; HIV = Human Immunodeficiency Virus; HCV = Hepatitis C Virus. ^a^Note that 88% of the calibration sample were current undergraduate students and 58 percent spoke English as a second language; estimated IQ therefore likely under-represents true intellectual function. ^b^n = 287. ^c^n = 292. ^d^n = 293.*

### Measures

#### Cognition

Both participant groups completed measures of decision making (IGT) and estimated premorbid intellectual ability (Wechsler Test of Adult Reading; WTAR; Wechsler, 2001). We assessed decision making with a computerized version of the IGT (see Appendix for details of payoff structure). IGT performance was characterized in terms of Net score (expressed as the proportion of cards selected from the advantageous decks) over all trials and in the last 60 trials after the initial exploratory phase (Brand et al., 2007). As recommended by Steingroever et al. (2013a), we also tabulated the proportion of cards selected from each deck in order to reveal potential differences in deck preference related to frequency of losses. Learning curves were assessed using the common practice of comparing the proportion of cards selected from the advantageous decks across five blocks of 20 consecutive trials.

Verbal learning and memory was assessed in primary analyses (see below) with the Hopkins Verbal Learning Test Revised^3^ (HVLT-R; Brandt and Benedict, 2001). Additional neurocognitive measures were used to describe cognitive functioning of the community sample. Measures included those capturing complex attention (Stroop Color and Word Test; Golden and Freshwater, 2002), reversal learning (CANTAB Intradimensional-extradimensional shift task; IDED; Fray et al., 1996), and sustained attention (CANTAB Rapid Visual Information Processing Task; RVIP; Fray et al., 1996). Cognitive testing was conducted by trained research assistants supervised by a psychologist (AET) as detailed in prior reports of largely overlapping samples (e.g., Gicas et al., 2014).

#### Diagnosis, Traits, and Functioning

In the community sample, DSM-IV diagnoses were made by a psychiatrist (WGH and FV-R) through consensus with the Best Estimate Clinical Evaluation and Diagnosis (Endicott, 1988) using all available information. Information referred to for diagnostic purposes included Axis I symptoms (Mini International Neuropsychiatric Interview; Sheehan et al. (1998); Beck Depression Inventory 2nd Edition; Beck et al., 1961), personality disorder symptoms (International Personality Disorder Examination – Screener; Loranger et al., 1997), psychotic symptoms (Positive and Negative Syndrome Scale [PANSS]; Kay et al., 1987; 30-item full PANSS administered annually and short 5-item version administered monthly), and mental status (examination by a psychiatrist). Sensation seeking was measured with the Sensation Seeking Scale – Form V (SSS-V; Zuckerman, 1994), with a small number of missing items (<1%) imputed using expectation-maximization. Everyday functioning was indexed with the Role Functioning Scale, which incorporates functioning in the areas of work productivity, independent living/self-care, social relationships and community involvement (Goodman et al., 1993). The Global Assessment of Functioning captured psychiatric symptoms along with social and occupational functioning (American Psychiatric Association, 1994).

#### Health-Risk Behaviors

A *Health-risk Index* was the primary outcome. This measure incorporated substance use data as well as needle-sharing, pipe-sharing, injection drug use, and unprotected sex. Substance use data was collected monthly using the Time Line Follow Back method (TLFB; Sobell et al., 1986)^4^ except tobacco use data^5^, which was collected every 6 months.

Reported health-risk behaviors are summarized in Table 2, with scores contributing to the Index of Health-risk behavior in italics. To construct the Health-risk Index, substance-related “harm scores” representing physical, psychological, and social harms to the user were calculated. These scores reflect harms from the specific substances used^6^ that are weighted by use frequency. Specifically, monthly substance-related harm scores (harm to self) were calculated for each participant by multiplying the harm index of each substance by the mean days of use of that substance per month, then summing across all substance types (Nutt et al., 2010; Jones et al., 2013). These scores were aggregated and averaged (mean) for each participant across the months of data available during their first-year enrollment^7^ to create a single harm composite score. This approach stabilizes substance use variation originating from factors irrelevant to risk taking (e.g., weather, substance use market costs, and product availability). Additionally, the Maudsley Addiction Profile (MAP; Marsden et al., 1998), administered monthly, contributed to the Health-risk Index. This interview captured self-reported needle-sharing, injection drug use, and unprotected sex, and was supplemented with a question concerning sharing of crack pipes (carrying risk of HCV transmission). These four variables were dichotomized (i.e., zero instances or at least one instance of the behavior over the first year of the study^8^) and summed to produce a total score from 0 to 4, reflecting the number of behaviors impacting health reported throughout the first year of the study. Finally, we calculated the Health-risk Index (*z*-scores) by standardizing the mean of the substance-related harm score and the MAP health behaviors score, which showed a reasonably strong association in this sample (*r* = 0.39, *p* < 0.001).

**TABLE 2:** Reported rates of Health Risk Behaviors in the community sample.

Measure	n	Median (IQR)	Observed range	Possible range	Percent of n reporting any occurrence
**MAP number of health-risk activity types in one year**	294	2 (1)	0 – 4	0 – 4	91.80
Inject with used needle					5.40
Sex without condom					35.40
Smoke from shared pipe					61.60
Inject non-prescribed drugs					62.20
**Days of use, per 28 days (harm index in parentheses)**	294				
Tobacco (37.3)		28.00 (1.92)	0 – 28.00	0 – 28.00	92.30
Alcohol (56.1)		0.54 (3.34)	0 – 28.00	0 – 28.00	79.20
Crack cocaine (79.5)		4.00 (13.85)	0 – 28.00	0 – 28.00	76.80
Cannabis (25.3)		1.82 (15.60)	0 – 28.00	0 – 28.00	73.00
Heroin (73.0)		0.00 (4.23)	0 – 28.00	0 – 28.00	49.50
Powder cocaine (42.4)		0.00 (1.90)	0 – 26.75	0 – 28.00	44.00
Methadone (prescribed; 24.9)		0.00 (24.2)	0 – 28.00	0 – 28.00	43.10
Methamphetamine (68.8)		0.00 (1.57)	0 – 26.00	0 – 28.00	43.00
Amphetamine (40.8)		0.00 (0.00)	0 – 11.00	0 – 28.00	8.90
Methadone (non-prescribed; 24.9)		0.00 (0.00)	0 – 20.11	0 – 28.00	5.50
Ecstasy (18.5)		0.00 (0.00)	0 – 1.55	0 – 28.00	5.10
LSD (15.0)		0.00 (0.00)	0 – 0.17	0 – 28.00	1.40
GHB (37.9)		0.00 (0.00)	0 – 0.38	0 – 28.00	1.40
Ketamine (28.9)		0.00 (0.00)	0 – 0.33	0 – 28.00	1.00
**Total substance-related harm score^*a*^**		2562.26 (1494.85)	0 – 8353.21	≥0	

*Scores contributing to the Health-risk Index are in italics. MAP = Maudsley Addiction Profile. ^a^Total substance-related harm score was calculated for each participant as harm index multiplied by mean days of use per month, summed across all substance types.*

For the exploration of associations between IGT parameters and the use of specific substances, we coded frequency of use from 0 to 3 for each substance to address the non-normality of frequency distributions. This coding system aimed to divide participants into four groups: non-users of that particular substance, and three groups of approximately equal size of low-, medium-, and high-frequency users. A score of zero was assigned to all participants who reported no use of the substance in the first year of the study. The remaining participants were rank-ordered according to how frequently they used the substance, and the bottom one-third were assigned a score of 1, the middle one-third were assigned a score of 2, and the top one-third were assigned a score of 3^9^.

### Computational Modeling of IGT Performance

To ensure use of the best-fitting version of the PVL model for the current data set, we evaluated 2 learning rules and 2 choice rules (Busemeyer and Stout, 2002; Ahn et al., 2008; Fridberg et al., 2010). Equations for all four models are presented in the Appendix.

### Statistical Analyses

#### Parameter Estimation

For each of the four model versions, we estimated the parameters for each individual participant using Hierarchical Bayesian Analysis (HBA), which is more accurate and robust than maximum likelihood estimation (Wetzels et al., 2010; Ahn et al., 2011; Lee, 2011). The parameters were estimated separately for the community and the calibration samples, because the two groups were expected to have different mean parameter values. Markov Chain Monte Carlo sampling was performed using Hamiltonian Monte Carlo implemented by Stan Development Team (2018) via the Rstan interface for R. We modified the HBA package (Ahn et al., 2017) on github^10^ adding the trial dependent models and replacing the default Cauchy priors on population level variance parameters with Inverse Gamma priors to avoid unrealistically large prior variances^11^.

Individual participants’ parameter values were modeled as independent normal distributions, truncated to limit values to the appropriate range (as in Vassileva et al., 2013). The group-level parameter means were drawn from uniform distributions and the group-level variances (σ^2^) were modeled as inverse gammas with prior shape and scale of 5 and 1, respectively (as in Vassileva et al., 2013). Four independent MCMC chains were run each with 75,000 iterations after discarding 25,000 as burn-in. Convergence was determined by inspection of trace plots and confirmed by Gelman-Rubin test R-hat values at or very close to 1.00 (Gelman et al., 2004). Consequently, the chains were combined, and analysis was performed on the resulting 300,000 iterations.

#### Model Comparisons

We compared the Deviance Information Criterion (DIC; Spiegelhalter et al., 2002), a Bayesian model selection criterion, across models. Smaller DIC values indicate better model fit, relative to other models fitted to the same data. The DIC balances model fit with a measure of complexity approximating the effective number of parameters in the model. The DIC performs best when the number of observations is much larger than the effective number of parameters. In all of our cases, the number of observations was more than 2 orders of magnitude larger than the effective number of parameters. When using the DIC, if multimodality is sufficiently problematic a posterior expectation is not a reasonable point estimator. This concern was alleviated by replacing Cauchy priors on population level variance parameters with a more realistic description of our prior uncertainty through an Inverse Gamma.

#### Hypothesis Tests

We tested the hypothesized associations of posterior IGT parameters with health-risk behaviors (Hypothesis 1) and learning and memory (Hypothesis 2) by conducting multivariate Bayesian linear regressions on community sample data. Rather than identifying a single “best” value for the models’ parameters, the aim of these regressions was to determine the posterior distribution for the models using an approach that accommodates poorly distributed data. The posterior distribution tells us how plausible each parameter value is, given the data collected. The Bayesian approach allowed us to propagate the distribution of PVL parameters into the regression model. The full posterior for the PVL parameters was then directly used as independent variables to the regression model. Each MCMC iteration proceeded as a Gibbs sampler, in which we drew a set of PVL parameters from their posterior and then drew the regression parameters. By sampling the PVL parameters and then feeding them into the regression models in independent MCMC runs, we used the same PVL parameter distributions for all of our regression models. Although performing all posterior sampling for the PVL parameters and the regressions in a single MCMC is possible, it would have muddled inference by introducing dependencies between regression models.

For the test of whether lower attention to losses is associated with more health-risk behaviors (i.e., Hypothesis 1), we ran Bayesian regression analyses controlling for demographics correlated with risk behaviors (*p* < 0.10; age and gender) since demographics might obscure findings, e.g., younger participants may have more opportunities to engage in behaviors by virtue of having different peer groups or lifestyles. We incorporated age and gender into the multivariate Bayesian linear regression using Markov Chain Monte Carlo simulation and the Metropolis-Hastings algorithm (using the $$$“MCMC” package implemented in R^12^; Martin et al., 2011). A Gelman-Rubin convergence diagnostic value of 1.02 was obtained based on 4 parallel chains, each with 37,500 iterations after discarding 12,500 iterations for burn in. The Gelman-Rubin diagnostic assesses differences in convergence between different MCMC runs, which were started from different parameter values and random seeds (Gelman and Rubin, 1992). Within each of the 4 parallel chains a Geweke diagnostic was used to ascertain that after discarding burn in iterations each chain was sampling from a stationary distribution (Geweke, 1989). For the regression variance term, we used an Inverse Gamma prior with shape and scale both equal to 3. This distribution has a mean of 1.5 and a variance of 2.25 reflecting our expectation of a well-fitting regression model. For the regression coefficients, improper uniform priors were used since we had sufficient data to overcome our uncertainty about parameter magnitude.

#### Exploratory Analyses

We used correlation analyses to explore associations between the IGT Net scores and the outcome variables (i.e., health-risk behaviors, learning and memory and sensation seeking). We present the results of these analyses with Bonferroni correction to address Type I errors. We also examined correlations between sensation seeking and health-risk behaviors (including partial correlations controlling for age and gender) and correlations between IGT parameter values and traditional IGT Net scores.

We employed Bayesian regression analyses to investigate associations between IGT parameters and the use frequency of individual substances, controlling for age and gender, which are often associated with the type of substances used (e.g., Conway et al., 2002). We also used Bayesian regression analysis to examine associations between IGT parameters and sensation seeking.

## Results

### Overall Cognitive Performance in Precariously Housed Community Participants

Despite estimated premorbid IQ falling within normal limits, cognitive functioning was impaired in the community sample as indicated by normatively derived *z*-score deviations, on measures of complex attention (RVIP A′ *M* = –1.19, *SD* = 1.28), verbal learning and memory (HVLT-R Immediate Recall *M* = –1.95, *SD* = 1.00), and non-verbal problem solving (IDED Stages *M* = –1.41, *SD* = 2.20). The exception was on a measure of response inhibition, which fell within normative expectations (Stroop Color Word Trial *M* = –0.08, *SD* = 0.93).

### Patterns of Decision Making in the Community and Calibration Samples

**FIGURE 1:**
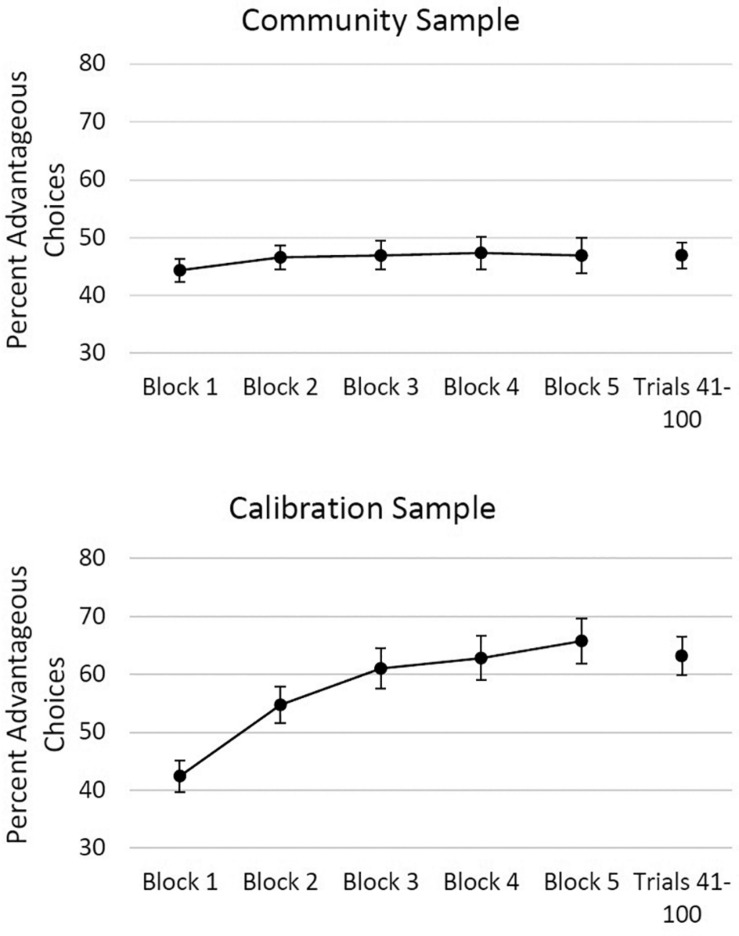
Iowa Gambling Task performance across five blocks of trials and in Trials 21 through 100, in the community **(top panel)** and the calibration sample **(bottom panel)**. Over 100 trials, percent advantageous scores of 30, 40, 50, 60, 70, and 80 are equivalent to Net scores of –40, –20, 0, 20, 40, and 60. Error bars represent 95% confidence intervals.

Figure 1 shows that the community sample demonstrated poor performance (IGT percent advantageous *M* = 46.31, *SD* = 14.83; last 60 trials *M* = 46.92, *SD* = 19.33) and a flat learning curve, whereas the calibration sample showed a significant improvement revealed by traditional IGT Scores across trial blocks (IGT percent advantageous *M* = 57.32, *SD* = 14.42; last 60 trials *M* = 63.16, *SD* = 19.27). Polynomial trends indicated no linear trend over blocks (i.e., no improvement of performance) in the community sample, whereas in the calibration sample a significant linear trend was observed (i.e., performance improved across blocks), *F*(1, 135) = 87.29, *p* < 0.001, along with quadratic, *F*(1, 135) = 17.72, *p* < 0.001, and cubic *F*(1, 135) = 4.89, *p* = 0.029, trends. The majority (55.5%) of community sample selected from Deck B more often than any other deck, whereas the most common favorite deck among the calibration sample was Deck D (46.5% of the calibration sample selected from Deck D more often than any other deck, while 30.9% selected most often from Deck B).

### Model Evaluation and Parameter Estimates in the Community and Calibration Samples

We selected the PVL model with the delta learning rule and the trial-dependent choice rule (PVL-Delta-TD) as the best fitting model for both samples, based on DIC values (see Table 3). Associations between traditional IGT Net scores and PVL model parameter estimates are described in Table 4.

**TABLE 3:** DIC values for the community and calibration samples indicating model fit for each model.

	DIC	
Model	Community sample	Calibration sample
PVL-Delta-TD	**–31461.25**	**–68394.44**
PVL-Delta-TI	–31210.48	–68102.44
PVL-Decay-TD	–31245.04	–65478.23
PVL-Decay-TI	–30271.01	–63966.25

*Values for the selected model are in boldface. Lower DIC values represent better fit compared to other models tested on the same data set. DIC values are group-level statistics. Delta = Delta learning rule; Decay = decay-reinforcement learning rule; TD = trial-dependent choice rule; TI = trial-independent choice rule.*

**TABLE 4:** Intercorrelations of traditional IGT Net scores and PVL-delta trial-dependent choice rule in 294 communiarticipants.

IGT Score or parameter	1	2	3	4	5
**Traditional IGT Net scores**					
(1) Total advantageous					
(2) Last 60 advantageous	0.941***				
**PVL-delta Trial-dependent choice rule**					
(3) Retention	**0.045**	**0.056**			
(4) Consistency	**–0.023**	**–0.014**	–0.128*		
(5) Attn to losses	0.756***	0.774***	0.074**	**–0.041**	
(6) Attn to magnitude	–0.415**	–0.390***	–0.191***	**–0.018**	–0.368***

Values in regular typeface are Pearson product-moment correlations; values in bold typeface are Spearman correlations (employed where skewness / standard error > 3.00). Shorter retention is reflected by larger values of the retention parameter. Total Advantageous = proportion of advantageous choices over all trials; Last 60 Advantageous = proportion of advantageous choices over the last 60 trials. *p < 0.05, **p < 0.01, and ***p <0 .001.

As illustrated in Table 5, the 95% confidence intervals for retention, attention to losses, and attention to magnitude did not overlap in the two samples. Interestingly, the mean consistency value was significantly greater than zero in the community and calibration samples, suggesting that on average, the alignment of choices with expected deck values increased over the course of the IGT task. Most remarkably, the community sample demonstrated minimal attention to losses, while the calibration sample weighted losses similarly to gains (attention to losses was not significantly different from 1.0).

**TABLE 5:** PVL model components of decision making in the community and calibration samples.

	Community sample (*N* = 294)		Calibration sample (*N* = 136)	
IGT Parameter (range)	Median (IQR)	95% CI	Median (IQR)	95% CI
Retention (0 - 1)	0.208 (0.280)	[0.261, 0.314]	0.169 (0.180)	[0.191, 0.250]
Consistency (-5 - 5)	0.353 (0.787)	[0.313, 0.439]	0.357 (0.712)	[0.289, 0.483]
Attention to losses (0 - 5)	0.192 (0.108)	[0.187, 0.241]	0.893 (0.842)	[0.941, 1.160]
Attention to magnitude (0 - 1)	0.556 (0.369)	[0.532, 0.601]	0.426 (0.281)	[0.412, 0.483]

*Shorter retention is reflected by larger values of the retention parameter.*

### IGT Component Processes, Health-Risk Behaviors, and Learning and Memory

Tables 6, 7 show the results of Bayesian linear regressions of community sample data. The mean represents the mean estimate of the posterior distribution, and the credible interval indicates the 95% probability that the coefficient falls within the described range. A 95% credible interval provides the upper and lower limits for the middle 95% of the distribution. That is, a 95% Bayesian credible interval provides a range for a parameter such that the probability that the parameter falls in that range is 95%. Wider intervals mean more uncertainty regarding the parameter. Credible intervals overlapping zero indicate uncertainty of an effect.

**TABLE 6:** Posterior summary of Bayesian linear regression for IGT PVL model parameters and Health Risk Behaviors, with and without demographic covariates.

		Health Risk Behaviors (*n* = 294)	
Model	Independent variable	Mean (SE)	95% Credible Interval
1	Age	–0.0163 (0.007)	[–0.030, –0.003]
	Gender	0.412 (0.161)	[0.096, 0.721]
	Retention	0.189 (0.247)	[–0.295, 0.677]
	Consistency	–0.124 (0.107)	[–0.335, 0.085]
	Attention to losses	–0.477 (0.375)	[–1.219, 0.260]
	Attention to magnitude	–0.115 (0.162)	[–0.430, 0.204]
2	Retention	0.156 (0.176)	[–0.187, 0.500]
	Consistency	–0.098 (0.111)	[–0.317, 0.122]
	Attention to losses	–0.430 (0.389)	[–1.206, 0.334]
	Attention to magnitude	–0.128 (0.170)	[–0.456, 0.194]

*Shorter retention is reflected by larger values of the retention parameter.*

**TABLE 7:** Posterior summary of Bayesian linear regression for IGT PVL model parameters and sensation seeking and learning and memory.

	Sensation seeking (*n* = 234)		Learning and Memory (HVLT-R; *n* = 281)	
Independent variable	Mean (SE)	95% Credible Interval	Mean (SE)	95% Credible Interval
Retention	0.101 (1.377)	[–2.593, 2.761]	4.081 (1.670)	[0.815, 7.338]
Consistency	0.589 (0.582)	[–0.566, 1.709]	1.089 (0.734)	[–0.341, 2.542]
Attention to losses	2.925 (2.097)	[–1.265, 6.984]	3.667 (2.544)	[–1.337, 8.618]
Attention to magnitude	–1.312 (0.909)	[–3.115, 0.456]	–1.242 (1.101)	[–3.388, 0.951]

*Higher SSS-V scores indicate higher sensation seeking; higher HVLT-R total recall scores indicate better learning and memory ability. Shorter retention is reflected by larger values of the retention parameter. Sixty participants did not complete the SSS-V.*

Table 6 reveals that younger age (post. *M* = –0.016) and female gender (post. *M* = 0.412) were associated with more health-risk behaviors. In terms of the Hypotheses, the null results indicated that lower attention to losses was unlikely to be associated with health-risk behaviors, regardless of whether demographics were controlled (Hypothesis 1; see Table 6). Our second hypothesis was also not supported: shorter retention (i.e., faster updating, indicated by larger retention parameter values) was associated with better learning and memory scores (Hypothesis 2; post. *M* = 4.081; see Table 7). Zero-order Bayesian regression failed to reveal associations between the IGT parameters values and use frequency of specific substances.

### Sensation Seeking and Risk Behaviors

Higher sensation seeking was associated with more health-risk behaviors (*sr*^2^ = 0.076 without demographic covariates, *sr*^2^ = 0.085 with age and gender as covariates). Table 7 indicates that the IGT parameters were not associated with sensation seeking.

### Traditional Net Score Associations

When Bonferroni correction was applied (*p* < 0.016 for three comparisons), the proportion of advantageous choices (i.e., IGT Net score) in the last 60 trials was not associated with health-risk behaviors. Higher Net scores were associated with better learning and memory (*r* = 0.16) and higher sensation seeking (*r* = 0.17).

## Discussion

To our knowledge, the current report is the first investigating links between decision making component processes derived from the IGT and health-risk behaviors in a large, inclusive sample of highly vulnerable community members. IGT performance in this sample is characterized by (a) very low attention to losses (versus gains) in an absolute sense and relative to the calibration sample, (b) increasing consistency (i.e., the probability of choosing the deck with the highest expected deck value increased across trials); further, relative to the calibration sample, both (c) high attention to the magnitude of outcomes, and (d) short retention of information across trials. Strikingly, the 95% confidence intervals for the retention, attention to losses, and attention to magnitude parameters were non-overlapping between the groups sampled, verifying the distinctiveness of IGT performance in this vulnerable community sample. As in previous studies, community participants with substance use disorders demonstrated a flat learning curve and low Net scores (i.e., low proportion of advantageous choices; Mukherjee and Kable, 2014). These observations suggest that the laboratory based IGT and its derived component processes diverge remarkably in two highly distinctive participant groups.

The community sample’s striking lack of attention to losses is a key observation of this research (also see Fridberg et al., 2010; Vassileva et al., 2013). This parameter can range from zero, where there is no attention to losses, up to five, where losses are weighted five times as important as gains (a value of 1 reflects equal weight given to losses and gains). We observed parameter values that ranged from 0.01 to 0.75 in the community sample, indicating little variability and a consistent failure to consider losses. In contrast, amongst our calibration sample, the attention to losses parameter ranged from 0.06 to 3.2. Importantly, the remarkable insensitivity to losses we observed suggests that in select highly vulnerable persons, losses on the IGT may not “register” under these laboratory circumstances.

### Health-Risk Behaviors

Interestingly, female gender was associated with more engagement in health-risk behaviors in this sample. Further, contrary to our hypothesis, there was no association between attention to losses on the IGT and health-risk behavior, despite our advanced computational modeling. Likewise, exploratory analysis failed to reveal associations between the IGT parameters and frequency of use of specific substances. The lack of IGT associations to health-risk behaviors was apparent in analyses using traditional Net scores as well. While focused primarily on substance use related health-risks, we identified another set of conditions (e.g., large sample of active substance users, marginalized persons, IGT parameter estimation) in which the ecological validity of the IGT appears limited (also see Gonzalez et al., 2005; Wardle et al., 2010). Importantly, we show that the IGT components, as extracted here, do not reveal unrecognized associations to select health-risk outcomes that could be masked in conventional Net score analysis.

### Verbal Learning and Memory

Results did not support an interpretation that longer retention of expected values would be associated with better learning and memory ability in this cognitively impaired community sample. Contrary to our hypothesis, shorter retention of expected values (more rapid updating) on the IGT was associated with better learning and memory. Our unsupported hypothesis was premised on the notion that rapid updating would reveal a myopic attentional focus at the expense of robust memory encoding that would emerge with longer retention of expectancies. However, persons who show slower updating of expectancies, with an accordingly more remote focus, showed poorer learning and memory. Apparently, individuals who update their expectancies less readily may “miss” emergent information, which is associated with poorer HVLT-R memory.

Notably, the retention parameter was not associated with overall IGT performance as indicated by traditional Net Scores. As would be expected, more advantageous choices on the last 60 trials of the IGT were associated with better learning and memory.

### Sensation Seeking

Sensation seeking was not associated with any of the IGT parameters. Furthermore, despite the lack of associations between the IGT and health-risk behaviors in our sample, we replicated the prior reports of an association between sensation seeking and health-risk behaviors (8.5% of the variance; see Wardle et al., 2010). The significant association observed between real world behaviors and self-reported sensation seeking is similar to stronger associations reported between self-report executive function *ratings* (compared to executive *tests*) in predicting real world behaviors, such as occupational function (Barkley and Murphy, 2010). This pattern of stronger links between self-report ratings and real world functioning has been argued to reflect the limited sampling of basic cognitive components captured by tests versus more compiled and strategic levels of function captured over longer timeframes by self-report (Barkley and Murphy, 2010).

Surprisingly, conventional Net scores suggestive of conservative decision making showed a positive but weak association with higher sensation seeking. Prior research indicates that the association between the sensation seeking and health related behaviors may be moderated by IGT capacities (Gonzalez et al., 2005). Specifically, in a sample of polysubstance using HIV+ and HIV- participants, the association between sensation seeking and engagement in sexual practices that convey health risks was limited to HIV+ participants with better IGT capacities. Such capacities have been discussed as a potential marker of a relatively intact affective decision making brain system (Gonzalez et al., 2005; Wardle et al., 2010), which the authors considered as a possible prerequisite for feeling “salience” for the decisions taken. While the current observations do not address these moderating IGT effects, the association between better Net Scores and sensation seeking may emerge because persons better able to discern risks on the IGT may also be slightly more likely to endorse items on the Sensation Seeking Scale that reflect greater levels of affective salience.

### Strengths and Limitations

We investigated a large sample of persons precariously housed in the community who suffer from substance use disorders. This sample represents the largest investigation to date of the extent to which decision making performance on the IGT indicates health-risk behavior in an exceedingly vulnerable population. We employed computational modeling and state-of-the-art HBA techniques to reveal the specific component processes of decision making. Our inclusive community sample promotes generalizability of the findings and enables investigations of behaviors related to ongoing substance use.

We propagated uncertainty in parameters in the PVL model into the linear regression model by using samples from the PVL posterior as independent variables. This approach allowed us to carry the uncertainty in the individual level parameters through to the linear regression model to test whether components such as attention to losses were associated with health-risk behaviors. The result is a whole range of inferential solutions, rather than a point estimate and a confidence interval as in classical regression and the results depend on the full distribution of posterior samples from the PVL model analysis.

Despite these strengths, several limitations should be noted. First, a risk environment with ever-present social and structural vulnerabilities might contribute to the null observations in the prediction of real-world risk behaviors. As opposed to our operationalization of risk behaviors from an “outsider” perspective, the primary risks appreciated by vulnerable persons in the community might involve matters of personal safety in the context of victimization, poverty, housing insecurity, availability and cost of particular substances, availability of clean needles, etc., which are not captured by the Health-risk Index (see Rhodes, 2002; Shannon et al., 2006). While select IGT components may be sensitive to real-world risks, detection of such associations might be optimized when real-world risks are *fully perceived and appreciated* as personally salient, e.g., portending tangible gains and/or losses to the particular participant. Our Health-risk Index does not establish that risks are personally salient. Further, we did not granularly evaluate or confirm participants’ real-world loss/gain sensitivities or the extent to which detection of these real-world loses and gains covaried with the corresponding IGT components. Yet, our hypotheses addressed the ecological validity of the IGT in portending health-risks that do entail large potentials for adverse consequences, including brain injury and death. Nonetheless, addressing the issue of risk *perception and salience* in vulnerable persons might be a productive target for the future.

Second, financial outcomes on the IGT were hypothetical, as is most often the case. Although in studies of healthy participants there have been no differences in Net scores when performance-based cash incentives have been provided compared to standard hypothetical rewards (Bowman and Turnbull, 2003; Carter and Pasqualini, 2004), in one study comparing the impact of reward type on performance across persons dependent on cocaine and healthy controls an interaction was detected (Vadhan et al., 2009). Specifically, in the hypothetical reward condition the cocaine users’ performance was poorer than the healthy controls’ performance, whereas in the cash incentive condition there was no significant difference in performance across the groups. Notably, this sample was substantially less ill than our community sample; however, further investigation of this phenomenon is warranted to determine whether performance could be substantially improved with use of cash rewards in more complex populations.

Third, health risk behaviors, as captured by the *Health-risk Index*, were self-reported and might have limited reliability, and/or been under-reported or inaccurate. Indeed, unknown measurement error of the operationalized aggregated risk outcome may attenuate the hypothesized association. However, the accuracy of self-reports is thought to be adequate. Even socially undesirable behaviors were reported frequently and urine drug testing, in a largely overlapping sub-sample of participants, indicated an acceptable concordance with self-reported use (*n* = 3267 observations; percent agreement ranging from 83.0 to 87.1; kappa = 0.62 - 0.68; Jones et al., 2020). Furthermore, the credibility of our composite Health-risk Index is supported by its associations with theoretically relevant indicators such as sensation seeking and younger age.

Fourth, this sample was recruited from four SRO hotels and a community court in one low-income neighborhood. While this is an ideal sample to evaluate links between IGT component processes and real-world health-risk behaviors, the results are apt to have generalization limited to similar populations and contexts. Future studies may benefit from investigating decision making processes across a range of risk environments and divergent vulnerable populations. Nonetheless, a clear strength of the current study is that participants are in a naturalistic environment. Unfortunately, by virtue of structural vulnerability, these persons are at considerable risk for adverse consequences based upon their decisions. Despite this circumstance, we did not detect the expected patterns supporting the ecological validity of the IGT.

Fifth, we only investigated high-risk behavior in a precariously housed, multimorbid population. It would be useful to comprehensively evaluate associations between laboratory-based decision making and risk behavior across a broader population, including persons who use harmful substances or engage in other health-risk behavior but have adequate housing, stable employment, and/or good health. High-risk behaviors that are prevalent across the broader population that entail immediate reward with a lower probability of a large negative consequence are particularly important to investigate. For example, research targets might include high-risk sports, excessive speeding in a motor vehicle and occasional excessive consumption of alcohol.

Finally, we evaluated several versions of the popular PVL model for fit; but these models were initially generated from healthy (Yechiam and Busemeyer, 2005) as opposed to clinical samples. Moreover, the use of computational modeling necessitates a single model to characterize the potentially divergent individual processing strategies. Although the PVL model has outperformed other models in several studies, critics have noted that the best-fitting model depends on how the task is performed (Steingroever et al., 2013b, 2014). This represents a substantial challenge, given the range of strategies that are commonly employed in laboratory-based decision making tasks (Steingroever et al., 2013b, 2016; Worthy et al., 2013). In order to succeed on the IGT (i.e., to make a majority of advantageous choices), participants must first learn about the risk profiles of each deck and then choose more cards from the advantageous decks. Early trials on the IGT are thought to measure decision making under uncertainty, as the outcomes associated with each deck are unknown, whereas later trials are intended to measure decision making under risk (Brand et al., 2007; Upton et al., 2011). This design maps well onto real-world learning by consequences, and yet persons with learning and memory impairments such as the community sample might not progress to decision making under risk. Although the PVL model currently has strong empirical support (Ahn et al., 2008; Fridberg et al., 2010), the quest for a better model that can capture diverse decision making strategies continues (e.g., Dai et al., 2015; Haines et al., 2018).

### Conclusion and Future Directions

Computational modeling represents a valuable tool to elucidate the primary underpinnings of complex decision making. Indeed, well-specified component processes may ultimately serve as endophenotypes for substance use disorders and thereby contribute to advances in genetic and neurobiological research (Gottesman and Gould, 2003). As the search for laboratory tasks and decision making models continues, elucidating the ecological validity of various approaches is crucial in preserving the clinical relevance of the research. For example, a newer IGT model with five parameters representing rate of reward learning, rate of punishment learning, attention to frequency of rewards and losses, perseverance, and memory decay has demonstrated promising initial findings linking specific parameters to self-reported gambling problems (Haines et al., 2018; Kildahl et al., 2020).

The IGT remains one of the most widely researched behavioral decision making tasks, which is now marketed for clinical use (Bechara, 2012). Yet poorer IGT performance has been observed without apparent compromise to real-world decision making, eroding confidence in the measure’s ecological validity (e.g., Dunn et al., 2006; Steingroever et al., 2013a). Concerns have been raised about the validity of scores when used on an individual basis, even when component processes are estimated (Buelow and Suhr, 2009; Wetzels et al., 2010; Humphries et al., 2015). Consistent with these concerns is the lack of association between attention to losses and health-risk behaviors in the structurally vulnerable population we evaluated here.

Yet the usefulness of the IGT in precisely defining individual differences in decision making is also apparent. We observed a striking lack of attention to losses in persons engaged in high levels of health-risk behaviors. While greater inattention to losses did not converge with higher engagement in behaviors that put health at greater risk, it is clear that vulnerable individuals with substance use disorders often lacked risk aversion on the IGT. Previous studies have found a clear association between select brain regions (such as the ventral medial prefrontal cortex) and IGT performance (Noël et al., 2006). It is possible that although IGT responses are related to neural circuits associated with decision making, these individuals’ environments and other individual factors might dictate the level of risk persons partake in, to a greater extent than a neurocognitive test of decision making.

The current findings have implications for interventions aimed at reducing adverse outcomes among persons with substance use disorders. Most importantly, we found that negative consequences had little impact on the IGT choices made in this vulnerable, multimorbid population. Participants focused on recent cards and attended predominantly to gains while ignoring losses. Despite the lack of association between derived parameters and health-risk behavior, the parameter pattern is consistent with a pronounced insensitivity to negative consequences. It may be that this corresponds to the ineffectiveness of punishments alone in preventing recidivism in substance-dependent persons (Chandler et al., 2009). In fact, reward-based contingency management has been identified in a meta-analysis of controlled studies as one of the most effective psychosocial interventions for substance use disorders (Cohen’s *d* = 0.58; Dutra et al., 2008). Furthermore, the risk environment is an important determinant of behaviors with high potential for adverse outcomes (McNeil et al., 2015). While policies and interventions must keep factors such as demographics, traits and decision making processes in view, the risk environment can and should be a direct target for intervention (Degenhardt et al., 2010).

## Data Availability Statement

The data used in this study cannot be made publicly available because individual participant data includes identifiers which are necessary for these analyses. Further, this data cannot be publicly shared due to potential privacy infringement and related ethical and legal obligations to participants as restricted by the REBs of the University of British Columbia and Simon Fraser University. Requests to access the datasets should be directed to AT, aethornt@sfu.ca.

## Ethics Statement

The studies involving human participants were reviewed and approved by the Clinical REB, University of British Columbia and the REB, Simon Fraser University. The participants provided their written informed consent to participate in this study.

## Author Contributions

HB, AT, and WH designed the study. FV-R, WP, OL, WH, and TB collected the data. HB, PJ, NW, and DC performed the data analysis. HB, DC, AT, PJ, AJ, WP, DL, KG, WL, CG, GM, AB, AR, RP, and WH interpreted the data. HB, AT, DC, and PJ drafted the final report. TB was the study coordinator. AT provided oversight and supervision. All authors read and approved the final draft.

## Conflict of Interest

WH has received consulting fees or sat on Advisory Boards for the Translational Life Sciences (TLS), AlphaSights, GuidePoint, Newron, *In silico*, AbbVie, and Otsuka and holds shares in TLS and AbCellera. AB has received consulting fees or sat on Advisory Boards for the Bristol-Myers Squibb, Eli Lilly, and Roche. AR has received Advisory Board fees from the Hofmann-La Roche. GM has received consulting fees or sat on paid advisory boards for Apotex, AstraZeneca, BMS, Janssen, Lundbeck, Otsuka, Pfizer, and Sunovion and also received fees for lectures sponsored by the AstraZeneca, BMS, Janssen, Otsuka, and Eli Lilly, and has received grants from the Janssen Pharmaceuticals. RP has received speaking and Advisory Board fees from the Janssen, Lundbeck, and Otsuka. The remaining authors declare that the research was conducted in the absence of any commercial or financial relationships that could be construed as a potential conflict of interest.

## Footnotes

1 i.e., history of schizophrenia, significant head injury, current psychosis or neuroleptic treatment, or low education.

2 Thirty-three community participants were not included for the following reasons: not understanding the goal of the IGT (*n* = 2), poor engagement in the IGT (e.g., falling asleep or not looking at the screen; *n* = 12), completing fewer than 60 IGT trials (*n* = 8), switching decks fewer than 10 times throughout the task (*n* = 10), or having a severe neurological disorder compromising the interpretation of health-risk outcome measures (*n* = 1). In the calibration sample, one participant who did not understand the goal of the IGT (due to low proficiency in the English language) and one participant who switched decks fewer than 10 times were not included. Note that participants who completed fewer than 60 trials were not included because they did not participate in at least one block of the decision making under risk phase (Brand et al., 2007) and participants who rarely switch decks are considered to be minimally responsive to the outcomes of the trials, suggesting poor engagement in the task.

3 Two participants did not complete the HVLT-R, and 11 participants’ HVLT-R scores were considered invalid due to poor engagement (*n* = 5), poor English literacy (*n* = 2), errors in test administration (*n* = 2), or hearing impairment (*n* = 2).

4 This self-report substance use data was concordant with urine drug testing with an acceptable level of concordance with self-reported use (*n* = 3267 observations; percent agreement ranging from 83.0 to 87.1; kappa = 0.62 - 0.68; Jones et al., 2020).

5 Coded as none = 0 days per month, occasional = 4 days per month, and daily = 28 days per month. Tobacco use is typically relatively stable and was therefore carried forward from each time point to the next.

6 Including alcohol, amphetamine, cannabis, powder cocaine, crack cocaine, ecstasy, GHB, heroin, ketamine, LSD, methadone, methamphetamine, and tobacco.

7 One participant had no substance use data. Mean number of months = 9.6, *SD* = 3.7. Three outliers with absolute-value z-scores greater than 3.29 were examined for accuracy and adjusted to the next-highest value to reduce influence.

8 Continuous variables (e.g., mean number of instances reported) were not used due to extremely skewed distributions. All participants had at least one month of data. Mean number of months = 8.6, *SD* = 4.0.

9 Of the 237 participants included, 227 completed all four measures of risk behavior affecting others, 9 completed three of the measures, and 1 completed two of the measures.

10 https://github.com/CCS-Lab/hBayesDM

11 https://github.com/iamdavecampbell/hBayesDM

12 https://cran.r-project.org/web/packages/mcmc/mcmc.pdf

## Appendix

### IGT Payoff Structure

The payoff structure of each deck is presented in Appendix Table A1; Deck A yields high payoffs on every trial that are frequently paired with even higher losses, generating net losses in the long run. Deck B yields high payoffs on every trial and once every ten trials, a very large loss, for net losses in the long run. Deck C yields smaller payoffs on every trial that are frequently paired with small losses, generating net gains in the long run. Deck D yields smaller payoffs on every trial and once every ten trials, a moderate loss, for net gains in the long run. As more cards are chosen from Decks A and B throughout the task, the net losses become larger at a rate of $150 per ten cards. For example, the first ten cards in Deck A yield a total net loss of $250, while the next ten cards in Deck A yield a total net loss of $400. A maximum of 60 cards can be chosen from each deck before it is “depleted.” In contrast, as more cards are chosen from Decks C and D throughout the task, the net gains become larger at a rate of $25 per ten cards (the first ten cards yield a net gain of $250).

**TABLE A1:** Outcomes of disadvantageous and advantageous IGT decks.

	Disadvantageous decks		Advantageous decks	
Outcome characteristic	A	B	C	D
Mean magnitude of net gains	$100.00	$123.52	$40.30	$61.94
Frequency of net gains	0.28	0.90	0.83	0.90
Mean magnitude of net losses	–$126.74	–$1736.67	–$14.00	–$245.00
Frequency of net losses	0.72	0.10	0.17	0.10
Mean outcome per selection	–$62.50	–$62.50	$31.25	$31.25

*Means are calculated over 60 trials (decks are “depleted” after 60 trials). Every trial generates gains and some trials also generate losses; net outcomes reflect gains minus losses. For example, 28% of Deck A selections result in net gains averaging $100, whereas 72% of Deck A selections result in net losses averaging -$126.74; in the long run, the mean outcome of selections from Deck A is -$62.50 per card.*

### Equations for the PVL Model

The four versions of the PVL model that were evaluated for the present study each employ a utility function, a learning rule, and a choice rule.

#### Prospect Utility Function

According to the prospect utility function, which is employed in all versions of the PVL model, the subjective value (i.e., worth or “utility”), *u(t)* of the net outcome *x(t)* on trial *t* is calculated based on the actual outcome (i.e., the amount gained or lost), the attention to magnitude parameter (α), and the attention to losses parameter (λ):

$$u\left(t\right)=x{\left(t\right)}^{\alpha }\;ifx\left(t\right)\geq 0.$$

$$u\left(t\right)=-\lambda {\left|x\left(t\right)\right|}^{\alpha }\;ifx\left(t\right)<0.$$

The attention to magnitude parameter, α, gives the shape of the utility function and ranges between 0 and 1. When α is close to 0, the magnitude of the outcome has little impact on its subjective value (i.e., all gains are valued equally and all losses are valued equally); when α is close to 1, the subjective value is directly proportional to the magnitude of the net loss or gain. In contrast, the attention to losses parameter, λ, indexes how much attention the participant pays to losses compared to gains. The attention to losses parameter ranges between 0 and 5; values below 1 reflect less attention to losses than gains, and values above 1 reflect greater attention to losses than gains.

#### Learning Rules

##### Delta learning rule

Using the Delta learning rule (Rescorla and Wagner, 1972), the expected value, *E*, for a given deck, *j*, only changes after that deck is chosen (i.e., expected values for the other three decks remain static on that trial). The expected value for the chosen deck is updated by a proportion (*A*) of the prediction error (i.e., the difference between the previous expected value and the subjective value of the obtained outcome):

$$E_{j}=E_{j}\left(t-1\right)+A\cdot \delta_{j}\left(t\right)\cdot \left[u\left(t\right)-E_{j}\left(t-1\right)\right].$$

The variable δ_*j*_(*t*) is a dummy variable equal to 1 if deck *j* was chosen on trial *t*, and otherwise equal to 0. *A* is the retention parameter, which describes the weighting of the most recent versus more distant past outcomes, and ranges between 0 and 1. When *A* is closer to 0, the most recent outcome has a low influence (compared to more distant outcomes) on the new expected value (i.e., expected values are more stable over time). When *A* is closer to 1, the most recent outcome has a high influence on the new expected value (with *A* = 1, the previous expected value and the prediction error of the most recent outcome are weighted equally). Thus, lower retention is reflected by larger values of *A*.

##### Decay-reinforcement learning rule

According to the decay-reinforcement learning rule, the expected value for all four decks decays toward zero on every trial (modeling degradation of memory over time). The rate of decay is given by the retention parameter, *A*, which ranges between 0 and 1. The expected value of the chosen deck is then updated by the subjective value of the obtained outcome:

$$E_{j}=A\cdot E_{j}\left(t-1\right)+\delta_{j}\left(t\right)\cdot u\left(t\right).$$

With this learning rule, lower retention (i.e., higher reliance on recent outcomes) is reflected by smaller values of *A*, in contrast to the Delta learning rule. Note that the decay-reinforcement learning rule allows for a higher weighting of the most recent outcome (e.g., with *A* = 0, the new expected value is determined entirely by the most recent subjective value), compared to the Delta learning rule, in which the previous expected value is always given at least as much weight as the prediction error of the most recently obtained outcome.

#### Choice Rule

In all model versions, the probability of choosing deck *j* on the next trial, denoted *Pr*[*D*(*t* + 1) = *j*], is described by a ratio-of-strengths rule (Luce, 1959), increasing with higher expected value for that deck and decreasing with higher expected values for other decks:

$$Pr\left[D\left(t+1\right)=j\right]=\frac{e^{\theta \left(t\right)\cdot E_{j}\left(t\right)}}{\sum_{j=1}^{4}\left(e^{\theta \left(t\right)\cdot E_{j}\left(t\right)}\right)}.$$

The probability function includes a sensitivity parameter, θ*(t)*, that reflects the trade-off between exploration of new options (more random choices) and exploitation of high expected values (less random choices).

##### Trial-independent choice rule

Using this rule, the sensitivity parameter is constant over all trials, and is given by:

$$\theta \left(t\right)=3^{c}-1.$$

The consistency parameter, *c*, is allowed to range between 0 and 5 (although in practice, values of *c* are rarely higher than 1.0). Higher values of *c* reflect choices that are more consistent with expected values, while lower values of *c* reflect choices that are more random.

##### Trial-dependent choice rule

The trial-dependent choice rule models change in consistency throughout the task. Using this rule, the sensitivity parameter is a function of the trial number:

$$\theta \left(t\right)={\left(t/10\right)}^{c}.$$

With this choice rule, *c* is allowed to range between –5 and 5 (although values of *c* are typically close to zero). Positive values of *c* indicate increasing sensitivity (more deterministic decisions) over time, and could represent an initial exploration phase of learning about all decks followed by an exploitation phase, where the best deck is consistently chosen.

## Abbreviations

HBA: Hierarchical Bayesian Analysis
HVLT-R: Hopkins Verbal Learning Test Revised
IGT: Iowa Gambling Task
MAP: Maudsley Addiction Profile
PVL: Prospect Valence Learning
PVL-Decay-TD: PVL model with decay-reinforcement learning rule and trial-dependent choice rule
PVL-Decay-TI: PVL model with decay-reinforcement learning rule and trial-independent choice rule
PVL-Delta-TD: PVL model with delta learning rule and trial-dependent choice rule
PVL-Delta-TI: PVL model with delta learning rule and trial-independent choice rule
REB: Reseach Ethics Board
SRO: Single-room occupancy.
